# Analysis of Surplus Cryopreserved Blastocysts in Fresh Donor Oocyte Cycles

**DOI:** 10.1001/jamanetworkopen.2025.6193

**Published:** 2025-04-21

**Authors:** Shelun Tsai, Xiaoyue Ma, Samantha Spring, Steven Spandorfer

**Affiliations:** 1 Ronald O. Perelman and Claudia Cohen Center for Reproductive Medicine, Weill Cornell Medical College, New York, New York; 2 Pacific Northwest Fertility and IVF Specialists, Bellevue, Washington; 3 Department of Population Health Sciences, Weill Cornell Medical College, New York, New York

## Abstract

**Importance:**

Excess cryopreserved embryos present financial, logistical, and ethical constraints on patients and fertility clinics that are further complicated by the recently evolving US judicial landscape that may alter the legal status of frozen embryos and limit disposition options. It is therefore important to consider limiting the number of supernumerary embryos created from fresh donor oocytes.

**Objective:**

To determine the optimal number of fresh donor oocytes to fertilize to achieve 1 live birth while minimizing supernumerary blastocysts.

**Design, Setting, and Participants:**

This cohort study used data from recipients of fresh donor oocytes at member clinics of the Society for Assisted Reproductive Technology Clinic Outcomes Report System (SART CORS) between January 1, 2016, and December 31, 2020. Data were analyzed between August 2023 and June 2024.

**Main Outcomes and Measures:**

The number of supernumerary blastocysts for a given number of fresh donor oocytes retrieved.

**Results:**

There were 9395 fresh donor oocyte recipients included. The median (IQR) oocyte donor age at oocyte retrieval was 26 years (24-28 years), and the median (IQR) patient age at cycle start was 42 years (38-45 years). The median (IQR) number of oocytes retrieved was 20 (14-28), fertilized oocytes (2 pronuclei [2PN]) was 11 (7-17), usable embryos was 6 (3-10), and supernumerary blastocysts was 5 (2-8). When patients were divided evenly into quartiles based on the number of fresh donor oocytes retrieved (14 or fewer, 15 to 20, 21 to 28, 29 or more), it was associated with a significant increase in the number of cryopreserved supernumerary blastocysts (median [IQR] blastocysts by quartile: 2 [1-4] vs 4 [2-7] vs 6 [3-9] vs 9 [4-14], respectively; *P* < .001). However, the live birth rate was lower only for those in the first quartile receiving 14 or fewer oocytes (50.8% [1207 of 2376] vs 64.1% [1497 of 2336] vs 67.9% [1631 of 2401] vs 67.9% [1550 of 2282]; *P* < .001).

**Conclusions and Relevance:**

In this retrospective cohort study of fresh donor oocyte cycles, the number of supernumerary blastocysts to achieve 1 live birth for a given number of fresh donor oocytes exposed to sperm was approximated, which may be valuable for counseling patients.

## Key Points

**Question:**

What is the optimal number of fresh donor oocytes to expose to sperm to maintain the chance of 1 live birth while minimizing supernumerary blastocysts?

**Findings:**

In this cohort study of a national assisted reproductive technologies database with 9395 fresh donor oocyte recipients, the number of supernumerary blastocysts dramatically increased with the number of fresh donor oocytes exposed to sperm. However, after 7 to 9 oocytes, there did not appear to be additional improvement in live birth rates.

**Meaning:**

These results suggest that fertility clinics and patients should consider limiting the number of fresh donor oocytes exposed to sperm to minimize the creation of excess embryos.

## Teaser

This cohort study of US adults in an assisted reproductive technologies database examines the optimal number of fresh donor oocytes to achieve live birth while minimizing supernumerary blastocysts.

## Introduction

Embryo cryopreservation is an essential component of the in vitro fertilization (IVF) process, allowing couples to store embryos for future pregnancies without undergoing another ovarian stimulation cycle. However, with the standard practice of fertilizing all mature oocytes retrieved and improvements in laboratory and vitrification techniques, there is an ever-growing accumulation of supernumerary embryos.^1,2^ Between 2004 and 2013, there were an estimated 400 000 assisted reproductive technology (ART) cycles in the US with nearly 2 million cryopreserved embryos, over half of which were not subsequently transferred.^2^ Disposition of excess embryos is a challenging decision-making process for patients and imposes financial, logistical, and ethical constraints on clinics faced with potential indefinite storage of embryos.^3,4,5,6,7^ Patients may also have moral and religious objections to creating excess embryos. Lastly, the US Supreme Court’s *Dobbs v Jackson Women’s Health Organization*^8^ decision overturning *Roe v Wade*^9^ as well as the Alabama Supreme Court’s *LePage v Center for Reproductive Medicine*^10^ ruling equating cryopreserved embryos to living children further complicate the situation and may potentially alter the legal status of surplus frozen embryos.

To address surplus cryopreserved embryos, 1 strategy to consider is limiting the number of oocytes exposed to sperm. A 2023 study by Correia et al^11^ proposes a model to determine the optimal number of oocytes to fertilize for patients to optimize live birth rates while minimizing supernumerary embryos. However, 1 key population was omitted from the model: patients receiving donor oocytes. These patients are potentially more likely to have surplus embryos from good prognosis donor oocytes, and an analysis of this population may assist fertility clinics and donor oocyte programs in determining the appropriate allocation of donor oocytes, a known limited resource.

A 2024 study^12^ evaluating 543 patients who received fresh donor oocyte cycles at a single academic institution between January 2012 and November 2022 reported dramatic increases in the average number of supernumerary blastocysts cryopreserved associated with an increase in the number of mature donor oocytes retrieved and exposed to sperm (1 vs 2 vs 3 vs 6 supernumerary blastocysts for 7 or fewer, 8 to 10, 11 to 14, and 15 or more oocytes exposed to sperm, respectively), although there were no differences in live birth rates between groups. However, these data only represent outcomes from 1 institution and may not be generalizable at the national level. Therefore, in this study, we evaluated a national database of ART cycles to determine the optimal number of fresh donor oocytes to fertilize to achieve 1 live birth while minimizing supernumerary blastocysts.

## Methods

### Study Design

This study was approved by the institutional review board at Weill Cornell Medicine, and was exempt from informed consent requirements because data were deidentified. The Strengthening the Reporting of Observational Studies in Epidemiology (STROBE) reporting guideline for cohort studies.

The data used for this study were obtained from the Society for Assisted Reproductive Technology Clinic Outcome Reporting System (SART CORS). Data were collected through voluntary submission, verified by SART, and reported to the Centers for Disease Control and Prevention (CDC) in compliance with the Fertility Clinic Success Rate and Certification Act of 1992 (Public Law 102-493). SART maintains Health Insurance Portability and Accountability Act–compliant business associate agreements with reporting clinics. In 2004, following a contract change with the CDC, SART gained access to the SART CORS data system for the purposes of conducting research. Over 90% of all ART cycles in the US are performed at SART-member clinics. SART annually selects up to 10 clinics, approximately 2.5% of SART clinics, for an on-site validation visit utilizing metrics and a masked selection process to identify outlier clinics. Medical records are reviewed during the validation visit to verify the designation, outcome, and reporting of cycles. Clinics with significant systematic reporting errors undergo data correction. Six primary metrics and 26 secondary metrics are used for clinic selection. The metrics include low prospective reporting for both egg retrieval cycles and total cycles, high live birth rates in the various age groups, low cancellation rate, high percentage of total fertility preservation cycles, high percentage of embryo banking and oocyte banking cycles, high percentage of fertility preservation cycles where oocytes were thawed or embryos were transferred within a year, high percentage of deleted cycles, high percentage of cycles converted from intrauterine insemination, and low percentage of cycles in which no embryos were suitable for transfer with and without preimplantation genetic testing (PGT). SART does not validate the accuracy of data entry fields such as gonadotropin dosage, number of oocytes retrieved, number of fertilized oocytes, number of embryos cryopreserved, PGT results, or demographic fields such age and diagnosis.

Patients undergoing their first fresh donor oocyte cycle between January 1, 2016, and December 31, 2020, were included in this study. Patients were excluded if they used a directed oocyte donor, shared donor oocyte cycle, reciprocal IVF, gestational carrier, donor embryos, PGT, or surgically retrieved sperm. The primary outcome was the number of supernumerary blastocysts for a given number of fresh donor oocytes retrieved. Supernumerary blastocysts were defined as the number of blastocysts remaining after the first live birth. If a patient did not have a live birth, supernumerary blastocysts were the number of blastocysts at the final transfer cycle. Secondary outcomes included the number of usable embryos and live birth rate for a given number of fresh donor oocytes retrieved. Usable embryos were defined as embryos that were transferred back to the patient’s uterus or cryopreserved as a blastocyst.

Patients were stratified into 4 quartiles based on the number of fresh donor oocytes retrieved. Group sizes were determined by maintaining similar sample sizes in each quartile. A secondary analysis was performed using more clinically applicable cutoffs based on a prior study at a single academic institution (7 or fewer, 8 to 10, 11 to 14, and 15 or more oocytes) to allow for comparison between the datasets.^12^ Another subanalysis was performed excluding patients who did not have a live birth and had 1 or more supernumerary blastocysts remaining to provide a more conservative estimate of excess cryopreserved supernumerary blastocysts.

### Statistical Analysis

The median and IQR for continuous variables and frequency and percentage for categorical variables were used to summarize the population characteristics of oocyte donors and recipients, the blastocyst development and pregnancy outcomes, and the blastocyst development and pregnancy outcomes in different numbers of oocytes retrieved. A Wilcoxon rank sum test or Kruskal-Wallis test was used to compare continuous outcomes among different groups, and a χ^2^ test was performed to compare categorical variables.

To explore if the recipients’ age and/or the donors’ age could estimate the proportion of oocytes retrieved that yield to useable blastocysts, random forest models were built, considering the proportion of useable blastocysts was skewed and multimodal. Donors’ age, recipients’ age, body mass index (BMI), gravidity, parity, race, recipients’ infertility diagnosis (ovulatory disorder, diminished ovarian reserve, primary ovarian insufficiency, tubal factor, uterine factor, endometriosis, male factor, and unexplained infertility), and mean numbers of oocytes retrieved were included as variables in the random forest models. Race was self-reported (Asian, Black or African American, Hispanic or Latino, White, other [American Indian or Alaska Native, Native Hawaiian or other Pacific Islander, or multiracial], and unknown), and was included as a study variable because previous studies have shown it to be relevant to IVF outcomes. Testing and training dataset were randomly assigned by 80% and 20% of the data.

To explore if the recipients’ age and/or the donors’ age could impact the number of embryos needed to result in 1 live birth, survival analyses were performed using the sum of total number of blastocysts transferred across a patient’s all treatment cycles as the time. Both the Cox proportional hazard models and accelerated-failure time models with log-logistic distribution were fit with the above set of variables. All *P* values were 2-sided with statistical significance evaluated at α < .05. All analyses were done in SAS version 9.4 (SAS Institute, Inc) and R version 4.3.2 (R Core Team 2023).

## Results

A total of 9395 fresh donor oocyte recipients were included. These patients underwent a total of 13 240 embryo transfer cycles. The median (IQR) oocyte donor age at oocyte retrieval was 26 years (24-28 years); median (IQR) patient age at cycle start was 42 years (38-45 years) (Table 1). The vast majority of patients were nulliparous (6952 of 9293 [74.8%]). Ovulatory disorder (7720 of 9395 [82.2%]) and diminished ovarian reserve (7401 of 9395 [78.8%]) were the most common infertility diagnoses. A total of 459 patients (4.9%) had primary ovarian insufficiency.

**Table 1.** Patient and Cycle Characteristics for Patients Undergoing Their First Fresh Donor Oocyte Cycle, 2016-2020

Characteristics	Fresh donor oocyte recipient, No. (%) (N = 9395)
Donor oocyte age, median (IQR), y (n = 9164)	26 (24-28)
Patient age	
Median (IQR), y	42 (38-45)
<35 y	1230 (13.1)
35-37 y	962 (10.2)
38-40 y	1535 (16.3)
41-42 y	1511 (16.1)
>42 y	4157 (44.3)
BMI, median (IQR) (n = 7473)	25.1 (22.3-29.6)
Gravidity (n = 9299)	
0	4094 (44.0)
1	2054 (22.1)
2	1411 (15.2)
≥3	1740 (18.7)
Parity (n = 9293)	
0	6952 (74.8)
1	1578 (17.0)
2	435 (4.7)
≥3	328 (3.5)
Infertility diagnosis	
Ovulatory disorder	7720 (82.2)
Diminished ovarian reserve	7401 (78.8)
Primary ovarian insufficiency	459 (4.9)
Tubal factor	650 (6.9)
Uterine factor	504 (5.4)
Endometriosis	521 (5.6)
Male factor	1472 (15.7)
Unexplained infertility	366 (3.9)
No. oocytes retrieved, median (IQR)	20 (14-28)
No. fertilized oocytes (2PN),^a^ median (IQR)	11 (7-17)
No. usable embryos, median (IQR)	6 (3-10)
No. supernumerary blastocysts, median (IQR)	5 (2-8)
No. embryos transferred per transfer cycle	
0	2 (<0.1)
1	9129 (69.0)
2	4025 (30.4)
≥3	84 (0.6)
Day 3 embryo transfer	639 (4.8)
Day 5 embryo transfer	12287 (92.8)
No. patients with live births	5885 (62.6)
No. embryo transfers resulting in a live birth	6589 (49.8)

Abbreviation: BMI, body mass index (calculated as weight in kilograms divided by height in meters squared).

^a^ 2 pronuclei.

The median (IQR) number of oocytes retrieved was 20 (14-28), fertilized oocytes (2 pronuclei [2PN]) was 11 (7-17), usable embryos was 6 (3-10), and supernumerary blastocysts was 5 (2-8) (Table 1). Patients were divided evenly into quartiles based on the number of fresh donor oocytes retrieved (14 or fewer, 15 to 20, 21 to 28, 29 or more) (Table 2). With increasing numbers of fresh donor oocytes retrieved, the number of cryopreserved supernumerary blastocysts increased significantly (median [IQR] by quartile, 2 [1-4] vs 4 [2-7] vs 6 [3-9] vs 9 [4-14], respectively; *P* < .001). The live birth rate was lower for those in the first quartile who received 14 or fewer oocytes (50.8% [1207 of 2376 patients] vs 64.1% [1497 of 2336 patients] vs 67.9% [1631 of 2401 patients] vs 67.9% [1550 of 2282 patients]; *P* < .001).

**Table 2.** Association Between the Number of Fresh Donor Oocytes Retrieved and Embryo Development by Evenly Divided Quartiles

Embryo development	Median (IQR)				*P* value
	≤14 Oocytes retrieved (n = 2376)	15-20 Oocytes retrieved (n = 2336)	21-28 Oocytes retrieved (n = 2401)	≥29 Oocytes retrieved (n = 2282)	
Oocytes retrieved	11 (8-13)	17 (16-19)	24 (22-26)	35 (31-42)	<.001
Fertilized oocytes^a^	7 (5-9)	11 (8-13)	15 (11-18)	20 (14-26)	<.001
Usable embryos	3 (2-5)	6 (4-8)	7 (5-11)	10 (6-15)	<.001
Supernumerary blastocysts	2 (1-4)	4 (2-7)	6 (3-9)	9 (4-14)	<.001
Total patients with live births, No. (%)	1207 (50.8)	1497 (64.1)	1631 (67.9)	1550 (67.9)	<.001

^a^ 2 pronuclei.

We performed a secondary analysis using 7 or fewer, 8 to 10, 11 to 14, and 15 or more fresh donor oocytes as cutoffs based on prior data published by Spring et al^12^ from a single academic institution (Table 3). Even with these lower numbers of donor oocytes fertilized, similar findings were observed; the number of supernumerary blastocysts increased from median (IQR) of 1 (0-2) to 2 (1-4) to 3 (2-5) to 6 (3-10) blastocysts across the 4 groups (*P* < .001). Again, the live birth rate was significantly lower only for those in the first quartile receiving 7 or fewer fresh donor oocytes (29.2% [161 of 551 patients] vs 53.4% [307 of 575 patients] vs 59.1% [739 of 1250 patients] vs 66.7% [4678 of 7019] in each quartile, respectively; *P* < .001). If we consider each number of oocyte retrieved sequentially, the live birth rate begins to plateau at 7 to 9 oocytes retrieved, corresponding to when the number of supernumerary embryos began to significantly increase (Figure).

**Table 3.** Secondary Analysis of Association Between the Number of Fresh Donor Oocytes Retrieved and Embryo Development by Quartiles^a^

Embryo development	Median (IQR)				*P* value
	≤7 Oocytes retrieved (n = 551)	8-10 Oocytes retrieved (n = 575)	11-14 Oocytes retrieved (n = 1250)	≥15 Oocytes retrieved (n = 7019)	
Oocytes retrieved	6 (4-6)	9 (8-10)	13 (12-14)	24 (19-31)	<.001
Fertilized oocytes^b^	4 (2-5)	6 (5-7)	8 (6-10)	14 (10-19)	<.001
Usable embryos	2 (0-3)	3 (2-5)	4 (3-6)	7 (4-11)	<.001
Supernumerary blastocysts	1 (0-2)	2 (1-4)	3 (2-5)	6 (3-10)	<.001
Total patients with live births, No. (%)	161 (29.2)	307 (53.4)	739 (59.1)	4678 (66.7)	<.001

^a^ Quartiles determined by prior data with lower numbers of donor oocytes fertilized in Spring et al.^12^

^b^ 2 pronuclei.

**Figure.**
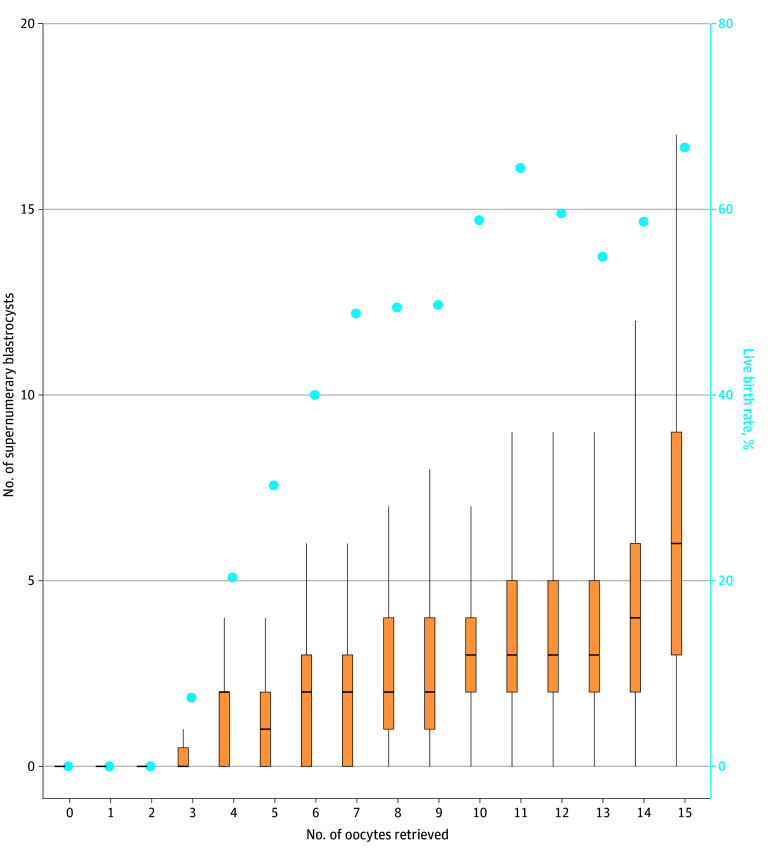
Association Between Number of Donor Oocytes and Number of Supernumerary Embryos and Live Birth Rate Center tick indicates median value; boxes, IQR; and whiskers, range. The number of supernumerary embryos increased as the number of fresh donor oocytes retrieved increased. The increase in supernumerary embryos was particularly significant after 7 to 9 fresh donor oocytes were retrieved, which also corresponded to when the live birth rate began to plateau.

Comparing patients who had live births with those who did not have a live birth from the set of fresh donor oocytes received, those with a live birth had a median (IQR) of 6 (3-9) supernumerary blastocysts remaining, with 5773 of 5885 patients (98.1%) having at least 1 supernumerary blastocyst remaining. In contrast, those without a live birth had a median (IQR) of 3 (1-7) supernumerary blastocysts remaining (*P* < .001), with 2810 of 3510 patients (80.1%) having at least 1 supernumerary blastocyst remaining (*P* < .001).

We performed a subanalysis excluding patients who did not have a live birth but had 1 or more supernumerary blastocysts remaining to provide a more conservative estimate of cryopreserved supernumerary blastocysts. A total of 6585 fresh donor oocyte recipients who underwent 9748 embryo transfer cycles were included in this subanalysis. The results were similar; the number of fresh donor oocytes retrieved was associated with an increase in the number of cryopreserved supernumerary blastocysts (median [IQR] by quartile: 2 [0-4] vs 5 [2-7] vs 7 [4-10] vs 9 [5-14], respectively; *P* < .001) (Table 4).

**Table 4.** Association Between Number of Fresh Donor Oocytes Retrieved and Embryo Development Excluding Patients Who May Potentially Return to Use Embryos

Embryo development	Median (IQR)				*P* value
	≤14 Oocytes retrieved (n = 1624)	15-20 Oocytes retrieved (n = 1609)	21-28 Oocytes retrieved (n = 1724)	≥29 Oocytes retrieved (n = 1628)	
Oocytes retrieved	11 (8-13)	17 (16-19)	24 (22-26)	35 (31-42)	<.001
Fertilized oocytes (2PN)^a^	7 (5-9)	11 (9-14)	15 (11-18)	21 (15-26)	<.001
Usable embryos	4 (1-5)	6 (4-8)	8 (5-11)	10 (6-15)	<.001
Supernumerary blastocysts	2 (0-4)	5 (2-7)	7 (4-10)	9 (5-14)	<.001
Total patients with live births, No. (%)	1207 (74.3)	1497 (93.0)	1631 (94.6)	1550 (95.2)	<.001

^a^ 2 pronuclei.

In assessing the association of usable blastocysts with donor and patient age, we evaluated the proportion of oocytes retrieved that yielded usable embryos. The median (IQR) estimated proportion was 0.32 (0.30-0.35), with minimal variation based on donor oocyte age and patient age (age 23 years or younger, 0.31 [0.29-0.35] vs age 28 years and older, 0.33 [0.30, 0.35]; younger than age 35 years, 0.30 [0.29-0.31] vs older than age 42 years, 0.33 [0.30-0.36]). There was no significant difference between different age groups. Both donors’ age and recipients’ age were not contributing factors for the proportion of usable blastocysts. Additionally, when assessing the number of embryos needed to result in 1 live birth, the accelerated-failure time models were with better model fit than Cox proportional hazard models. However, neither donors’ age nor recipients’ age were significant contributing factors.

## Discussion

This study showed a dramatic increase in the number of surplus cryopreserved blastocysts as the number of fresh donor oocytes exposed to sperm increased. The vast majority (74.7%) of fresh donor oocyte recipients obtained at least 15 fresh donor oocytes, yet 1 live birth was achieved in approximately 62.6% of patients after 1 or 2 embryo transfers. These patients subsequently had 6 excess supernumerary blastocysts in storage after achieving 1 live birth. These results provide information to support patients and clinicians who want to limit the creation of surplus embryos from fresh donor oocytes in patients who desire one child.

Our study adds to the study by Correia et al^11^ by including patients receiving fresh donor oocytes. This is particularly important as these fresh donor oocytes are associated with young oocyte age and good prognosis—and thus, more surplus embryos. In addition, donor oocytes are a limited resource, and determining the optimal number of fresh donor oocytes to allocate and fertilize to maintain live birth rates while minimizing supernumerary blastocysts can assist in the design of infrastructure and policies for fertility clinics and donor oocyte programs.

These data should be taken in the context of individual patient desires and comfort regarding supernumerary blastocysts and live birth rates. For example, a patient who wants 1 child and wants to prioritize minimizing supernumerary embryos may consider fertilizing 7 fresh donor oocytes or less for a median of 1 surplus embryo created. On the other hand, a patient may prefer to fertilize 8 to 10 fresh donor oocytes to have a median of 2 unused embryos while obtaining a higher live birth rate. Based on our results, fertilizing over 15 fresh donor oocytes significantly increases the number of supernumerary blastocysts without improving the live birth rate.

In addition to providing data for patients with moral or religious concerns about creating excess embryos, these estimations and considerations are particularly relevant in light of the recently evolving issues surrounding the legal status of embryos in the US. In 2022, the US Supreme Court’s *Dobbs v Jackson Women’s Health Organization*^8^ decision overturned *Roe v Wade*,^9^ and in 2024, the Alabama Supreme Court’s *LePage v Center for Reproductive Medicine*^10^ decision equated cryopreserved embryos to living children and held clinics liable for accidental destruction of embryos. While the Alabama state legislature subsequently passed a bill protecting fertility clinics from liability for accidental embryo destruction, questions remain unanswered about whether cryopreserved embryos are potentially considered persons, the so-called “personhood” definition based on “life beginning at fertilization.” Such determination will inevitably raise questions about the legality of embryo disposition options. Currently, embryo disposition options typically include discarding, donating to another couple or person, or donating to research, but the legal landscape may result in limiting options to either donation to another couple or person or storing the embryos indefinitely. These remaining options may essentially force procreation or burden patients and clinics with impractical indefinite storage. Therefore, serious deliberation should be undertaken for limiting the number of donor oocytes exposed to sperm.

### Strengths and Limitations

Strengths of our study include the use of a large, national database (SART CORS), which encompasses over 90% of fertility clinics and provides a generalizable analysis. However, as with all studies utilizing national databases, there are limitations surrounding the lack of nuanced data on the individual level. The number of oocytes retrieved, as documented in SART CORS, does not specify whether the oocytes were mature or immature. Analysis based on the number of mature oocytes would be more helpful than including all oocytes retrieved. Additionally, there is no information regarding the reasons underlying why patients had supernumerary blastocysts and whether they were potentially returning later to use them. Our analysis was also limited to obtaining 1 live birth, while, in reality, patients may ideally desire more than one child. Lastly, further studies on the outcomes of frozen donor oocytes should be evaluated to complete this counseling resource, as the chance of supernumerary blastocysts and live births with a certain number of frozen donor oocytes will be a consideration if only a portion of fresh donor oocytes are fertilized and the remaining are cryopreserved as oocytes.

## Conclusions

In this study, the number of fresh donor oocytes retrieved was positively associated with the number of supernumerary blastocysts, but the live birth rate was lower only among those who received 14 or fewer oocytes. The political and legal environment surrounding the status of a cryopreserved embryo and embryo disposition options has been recently evolving and changing. Reduction of supernumerary blastocysts by identifying the optimal number of oocytes to fertilize to achieve 1 live birth can be an important consideration in this uncertain landscape. This study provides valuable counseling information for patients using fresh donor oocytes, helping to approximate the number of supernumerary blastocysts to achieve 1 live birth for a given number of fresh donor oocytes exposed to sperm.

## Notes

Supplement.
Data Sharing Statement
