# Supplementary material for: Analysis of Surplus Cryopreserved Blastocysts in Fresh Donor Oocyte Cycles
Source: JAMA Netw Open. 2025 Apr 21;8(4):e256193. doi: 10.1001/jamanetworkopen.2025.6193 (PMC12013347; doi:10.1001/jamanetworkopen.2025.6193)
Supplement: Supplement. — Data Sharing Statement [file jamanetwopen-e256193-s001.pdf]

Data Sharing Statement

Tsai. Analysis of Surplus Cryopreserved Blastocysts in Fresh Donor Oocyte Cycles. *JAMA Netw Open*. Published April 21, 2025. doi:10.1001/jamanetworkopen.2025.6193

Data
Data available: No

Additional Information
Explanation for why data not available: Data protection regulations prohibit the data from being shared publicly. However, the data is accessible to researchers affiliated with a SART member clinic.
